# *Methylomonassediminis* sp. nov., *Methylomonas paludicola* sp. nov., and *Methylomonas roseola* sp. nov.: genomic insights into the evolution of methane oxidation and carotenoid biosynthesis

**DOI:** 10.3389/fmicb.2026.1954800

**Published:** 2026-09-22

**Authors:** Byeong Jun Choi, Jeong Min Kim, Dae Seung Lee, Hui Seong Won, Jae Kyeong Lee, Dae Gyu Choi, Jong Seok Lee, Yoonyong Yang, Moonsuk Hur, Che Ok Jeon

**Affiliations:** 1 Department of Life Science, Chung-Ang University, Seoul, Republic of Korea; 2 National Institute of Biological Resources (NIBR), Incheon, Republic of Korea

**Keywords:** carotenoid biosynthesis, evolution, methane monooxygenase, Methylomonas, novel taxa

## Abstract

Three reddish-pink, gram-stain-negative, motile, flagellated, short rod-shaped methanotrophs, designated BW4-1^T^, SPW-1^T^, and PHL2-19^T^, were isolated from wetland sediments in South Korea. All three strains possessed intracytoplasmic membranes characteristic of type I methanotrophs and grew with methane or methanol as the sole carbon and energy source. Their major fatty acids were C_14:0_, C_16:0_, and summed feature 3 (C_16:1_*ω*7*c* and/or C_16:1_*ω*6*c*). Phylogenetic and phylogenomic analyses placed the strains within the genus *Methylomonas*, with *Methylomonas defluvii* OY6^T^ as their closest relative. Despite sharing 99.9–100.0% 16S rRNA gene sequence similarity, the strains were differentiated by genomic and phenotypic characteristics. Pairwise average nucleotide identity and digital DNA–DNA hybridization values among the three strains were 95.0–95.9% and 60.4–66.2%, respectively, supporting their recognition as distinct species. All three strains possessed complete *pmoCAB* and *mmoXYBZDC* clusters and pathways linking methane-derived carbon to C_30_ and C_40_ carotenoid biosynthesis. PmoA phylogeny supported predominantly vertical inheritance of *pmoCAB*, whereas the patchy distribution and phylogenetic incongruence of MmoX indicated lineage-specific gain, loss, and possible horizontal transfer of sMMO-associated genes. Ancestral reconstruction further indicated that pMMO-dependent methane oxidation and carotenoid biosynthesis were evolutionarily conserved, whereas the sMMO system was shaped by more dynamic, lineage-specific evolutionary processes. Accordingly, the names *Methylomonas sediminis* sp. nov. (BW4-1^T^ = KACC 24053^T^ = MCCC 1K10705^T^), *Methylomonas paludicola* sp. nov. (SPW-1^T^ = KACC 24052^T^ = MCCC 1K10729^T^), and *Methylomonas roseola* sp. nov. (PHL2-19^T^ = KACC 24054^T^ = MCCC 1K10706^T^) are proposed.

## 1 Introduction

Methane is a potent greenhouse gas with a global warming potential approximately 26–41 times greater than that of carbon dioxide, highlighting the need for effective mitigation strategies (Shindell et al., 2009). Aerobic methanotrophs oxidize methane as their primary carbon and energy source, contributing to methane removal from natural and engineered environments. Methanotrophs are traditionally classified into type I and type X lineages within the *Gammaproteobacteria* and type II lineages within the *Alphaproteobacteria*, which differ in cellular organization and carbon-assimilation pathways (Knief, 2015). Methane oxidation is initiated by particulate methane monooxygenase (pMMO), encoded by *pmoCAB*, or soluble methane monooxygenase (sMMO), encoded by *mmoXYBZDC*. Whereas pMMO is widely conserved, sMMO occurs only in certain species or strains and may enhance metabolic flexibility under copper-limited conditions (Oshkin et al., 2023; Kalyuzhnaya et al., 2025).

The genus *Methylomonas*, belonging to the family *Methylococcaceae* within the class *Gammaproteobacteria* of the phylum *Pseudomonadota*, was established with the description of *Methylomonas methanica* (Whittenbury and Krieg, 1984). Members of *Methylomonas* have been isolated from diverse environments, including ponds, freshwater creeks, subsurface habitats, sediments, estuaries, manure, and rice paddies (Ogiso et al., 2012; Hoefman et al., 2014; Bussmann et al., 2021; Tikhonova et al., 2023; Abin et al., 2024; Gwak et al., 2025). They are generally Gram-stain-negative, strictly aerobic, short rods possessing the intracytoplasmic membranes characteristic of type I methanotrophs (Ouyang et al., 2024; Zhu et al., 2022).

Many *Methylomonas* species produce pink, red, or orange carotenoids, including 4,4′-diapolycopenoic acid and 4,4′-diapolycopene-4,4′-dioic acid (Oshkin et al., 2023; Tikhonova et al., 2023). Carotenoids may protect cells from oxidative stress and stabilize pMMO-containing intracytoplasmic membranes (Zhu et al., 2022). Their production from methane-derived carbon also has potential for sustainable synthesis of value-added compounds. However, the distribution and evolution of C_30_ and C_40_ carotenoid biosynthetic pathways in *Methylomonas* and related methanotrophs remain poorly understood, as do their evolutionary relationships with the broadly conserved pMMO and variably distributed sMMO systems.

In this study, three reddish-pink methanotrophs, BW4-1^T^, SPW-1^T^, and PHL2-19^T^, were isolated from wetland sediments and characterized using a polyphasic taxonomic approach. Their phylogenetic positions and genomic relatedness were evaluated, and the evolutionary histories of their methane monooxygenases were examined using PmoA- and MmoX-based phylogenies. Comparative genomic and ancestral gene gain-loss analyses were further conducted to reconstruct metabolic links between methane oxidation and C_30_ and C_40_ carotenoid biosynthesis and to investigate the evolution of these pathways. The resulting evidence supports the recognition of the three strains as novel species of *Methylomonas*.

## 2 Materials and methods

### 2.1 Enrichment and isolation of methanotrophic bacterial strains

To isolate methanotrophic bacteria, sediment samples were collected from wetlands in Hwaseong-si (37°15′44″N, 126°50′55″E) in October 2024 and at two sites in Gangneung-si (37°49′13″N, 128°53′13″E and 37°44′18″N, 128°57′13″E) in April 2025, Republic of Korea. For enrichment, the sediment samples were inoculated into 150-mL serum bottles containing 20 mL of fivefold-diluted modified nitrate mineral salts (NMS) medium (Whittenbury et al., 1970; Hoefman et al., 2012). Methane was added to constitute 20% (v/v) of the headspace, and the bottles were incubated at 25 °C for 2 weeks with gentle shaking. Subsequently, 10% (v/v) of each enrichment culture was transferred to fresh fivefold-diluted modified NMS medium and incubated under the same conditions for an additional 2 weeks. The enrichment cultures were then spread onto fivefold-diluted NMS medium solidified with 0.65% (w/v) agar, and the plates were incubated at 25 °C for 4 weeks under an atmosphere containing 20% (v/v) methane in air.

Colonies grown on the agar medium were screened for methanotrophic bacteria by PCR amplification of the *pmoA* and *mmoX* genes. The primer pairs pmoC374 (5′-AGC ARG ACG GYA CNT GGC-3′) and pmoA344 (5′-ANG TCC AHC CCA GAA GT-3′) were used to amplify *pmoA* (Ghashghavi et al., 2017), whereas mmoX534f (5′-CCG CTG TGG AAG GGC ATG AA-3′) and mmoX1393r (5′-CAC TCG TAG CGC TCC GGC TC-3′) were used to amplify *mmoX* (Horz et al., 2001). The 16S rRNA genes of *pmoA*- or *mmoX*-positive isolates were amplified using the universal primers 27F (5′-AGA GTT TGA TCM TGG CTC AG-3′) and 1492R (5′-TAC GGY TAC CTT GTT ACG ACT T-3′) (Lane, 1991), and the resulting amplicons were sequenced using primer 340F (5′-CCT ACG GGA GGC AGC AG-3′) (Muyzer et al., 1993). The resulting sequences were compared with those of type strains of species with validly published names using the EzBioCloud server (https://www.ezbiocloud.net/identify; Chalita et al., 2024). Based on these comparisons, three potentially novel *Methylomonas* strains, designated BW4-1^T^, SPW-1^T^, and PHL2-19^T^, were selected for further characterization. The strains were routinely cultured on NMS agar supplemented with 0.2% (v/v) methanol at 30 °C for 4 days and preserved at −80 °C in liquid NMS medium containing 15% (v/v) glycerol.

### 2.2 Phylogenetic analysis based on 16S rRNA gene sequences

The 16S rRNA gene amplicons of strains BW4-1^T^, SPW-1^T^, and PHL2-19^T^, initially generated using primers 27F and 1492R, were further sequenced using the universal primers 518R (5′-ATT ACC GCG GCT GCT GG-3′) (Muyzer et al., 1993) and 805F (5′-GAT TAG ATA CCC TGG TAG TC-3′) (Klindworth et al., 2013). Sequence reads generated using primers 340F, 518R, and 805F were assembled for each strain. Pairwise 16S rRNA gene sequence similarities among the three strains and closely related taxa were calculated using the nucleotide similarity search tool available on the EzBioCloud server. Multiple sequence alignments were generated using MUSCLE (Edgar, 2004) implemented in MEGA11. Phylogenetic trees were reconstructed using the maximum-likelihood (ML), neighbor-joining (NJ), and maximum-parsimony (MP) methods implemented in MEGA11 (Tamura et al., 2021). The nearest-neighbour interchange heuristic search, Kimura two-parameter model, and subtree-pruning-and-regrafting algorithm were applied for ML, NJ, and MP tree construction, respectively. The robustness of the resulting tree topologies was evaluated by bootstrap analysis based on 1,000 replicates.

### 2.3 Ecological distribution analysis based on 16S rRNA gene sequences

The potential ecological distributions of strains BW4-1^T^, SPW-1^T^, and PHL2-19^T^ were assessed using the Integrated Microbial Next-Generation Sequencing (IMNGS) platform (Lagkouvardos et al., 2016). Their 16S rRNA gene sequences were queried against 500,048 environmental 16S rRNA gene amplicon datasets using a sequence similarity threshold of 99.0%. These datasets represented diverse environments, including seawater, freshwater, marine and freshwater sediments, soil, plants, air, and host-associated microbiomes from oysters, corals, epibionts, and animal gastrointestinal tracts.

### 2.4 Genome sequencing, genome-based phylogeny, and genomic relatedness analyses

Genomic DNA was extracted from strains BW4-1^T^, SPW-1^T^, and PHL2-19^T^ grown in NMS medium supplemented with methanol using the Wizard Genomic DNA Purification Kit (Promega, USA), according to the manufacturer’s instructions. Whole-genome sequencing was performed using the Oxford Nanopore MinION platform (Oxford Nanopore Technologies, UK). The resulting reads were assembled *de novo* using Flye v2.9.6 (Kolmogorov et al., 2019), and genome completeness and contamination were assessed using CheckM2 v1.0.2 (Chklovski et al., 2023).

Phylogenomic relationships among the three strains and closely related taxa were inferred from the concatenated sequences of 120 single-copy bacterial marker genes (the bac120 set) identified using the Genome Taxonomy Database Toolkit (GTDB-Tk; v2.4.1) (Chaumeil et al., 2022) with reference to GTDB release R220. The concatenated sequences were aligned, and an ML tree was reconstructed in MEGA11 using the Jones-Taylor-Thornton (JTT) model (Jones et al., 1992), with nodal support evaluated by bootstrap analysis based on 1,000 replicates. Average nucleotide identity (ANI) and digital DNA–DNA hybridization (dDDH) values among the genomes of strains BW4-1^T^, SPW-1^T^, and PHL2-19^T^ and those of closely related type strains were calculated using the Orthologous Average Nucleotide Identity Tool (OrthoANI v0.93.1; www.ezbiocloud.net/tools/orthoani) (Lee et al., 2016) and the Genome-to-Genome Distance Calculator (GGDC v3.0; https://ggdc.dsmz.de/ggdc.php), respectively. dDDH values were estimated using formula 2 (Meier-Kolthoff et al., 2022).

### 2.5 Genomic characterization and phylogeny based on PmoA and MmoX

The genome sequences of strains BW4-1^T^, SPW-1^T^, and PHL2-19^T^ were deposited in GenBank. General genomic features and annotation data were obtained using the NCBI Prokaryotic Genome Annotation Pipeline (Tatusova et al., 2016). PmoA (particulate methane monooxygenase subunit A), MmoX (soluble methane monooxygenase component A *α*-subunit), and NifH (nitrogenase reductase) protein sequences from the three strains and closely related taxa were identified by BLASTP searches of their predicted proteomes using reference sequences obtained from the UniProt database.^1^ The identified PmoA and MmoX sequences were aligned using MUSCLE, and ML trees were reconstructed in MEGA11 using the JTT model. Nodal support was evaluated by bootstrap analysis based on 1,000 replicates.

### 2.6 Metabolic pathway reconstruction and gene gain-loss analysis

To minimize annotation bias, protein-coding genes in all genomes included in the comparative analysis were predicted using Prokka v1.14.6 (Seemann, 2014). The predicted proteins were functionally annotated using eggNOG-mapper v2 against the eggNOG v5.0 database with default parameters (Cantalapiedra et al., 2021). Metabolic pathways linking methane oxidation and assimilation to central carbon metabolism, isoprenoid precursor formation, and carotenoid biosynthesis were reconstructed based on KEGG Orthology identifiers and their corresponding Enzyme Commission numbers. Genes involved in these pathways were manually verified by BLASTN searches of the genomes of *Methylomonas*, *Methylobacter*, and *Methylomicrobium*, using homologous nucleotide sequences from closely related genomes as queries and an E-value threshold of <1 × 10^−5^.

To investigate genome evolution, orthologous gene families were identified from the predicted proteomes using OrthoFinder v2.5.5 with default parameters (Emms and Kelly, 2019). The resulting orthogroup presence-absence matrix was analyzed using COUNT (Csűrös, 2010), with the rooted GTDB-Tk phylogenomic tree used as the reference phylogeny. Ancestral gene repertoires and branch-specific gene gain and loss events were reconstructed using Wagner parsimony. The inferred gene content of each ancestral genome and the numbers of gene gains and losses along individual branches were mapped onto the phylogenomic tree. The distributions of genes encoding particulate methane monooxygenase (*pmoCAB*), soluble methane monooxygenase (*mmoXYBZDC*), and carotenoid biosynthesis enzymes (*crtE*, *crtM*, *crtN*, *crtP*, *crtNc*, *crtB*, and *crtI*) were also mapped across the analyzed genomes.

### 2.7 Physiological, phenotypic, and biochemical characterization

The temperature ranges for growth of strains BW4-1^T^, SPW-1^T^, and PHL2-19^T^ were determined on solid NMS medium supplemented with 0.2% (v/v) methanol at 4 °C and 10–45 °C in 5 °C increments for 4 days. The pH range for growth was determined in liquid NMS medium adjusted to pH 4.5–8.5 in 0.5-unit increments using 5 mM sodium citrate (pH 4.5–6.0) or sodium phosphate (pH 6.5–8.5) buffer. NaCl tolerance was assessed in liquid NMS medium containing 0–3.0% (w/v) NaCl in 0.5% increments for 4 days. Anaerobic growth was evaluated by incubating cultures on solid NMS medium at 30 °C for 21 days in a GasPak Plus system (BBL, USA).

Cell morphology and motility were examined by phase-contrast microscopy (Axio Scope.A1; Carl Zeiss, Germany). For detailed examination of cell morphology and flagella, cells were mounted on Formvar-coated copper grids, negatively stained with UranyLess (Electron Microscopy Sciences, USA) for 1 min (Benmeradi et al., 2015), and observed using a transmission electron microscope (JEM-F200; JEOL, Japan). Intracytoplasmic membrane structures were examined by transmission electron microscopy of ultrathin sections, following Ouyang et al. (2024) with modifications. Briefly, cells were prefixed overnight at 4 °C with 2% (w/v) glutaraldehyde and 2% (w/v) paraformaldehyde in 0.05 M sodium cacodylate buffer and then postfixed with 1% (w/v) osmium tetroxide for 1 h at room temperature. The fixed cells were incubated overnight at 4 °C in 0.5% (w/v) uranyl acetate and dehydrated through a graded ethanol series (30, 50, 70, 80, 90, and 100% [v/v]) for 20 min at each step. Dehydration with 100% ethanol was repeated three times. The samples were sequentially infiltrated with 1:1 and 1:2 mixtures of ethanol and resin for 1.5 h each, followed by incubation in 100% resin (Electron Microscopy Sciences) overnight at room temperature. The resin was replaced with fresh resin, and the samples were incubated for an additional 3 h before embedding and polymerization overnight at 70 °C. Ultrathin sections were prepared using an ultramicrotome (EM UC7; Leica, Germany) and sequentially stained at room temperature with 2% (w/v) aqueous uranyl acetate for 20 min and 3% (w/v) lead citrate for 5 min. The sections were examined using a JEM-1010 transmission electron microscope (JEOL) operated at an accelerating voltage of 80 kV.

Unless otherwise specified, the phenotypic characteristics of the three strains and the reference strain, *M. defluvii* KCTC 8159^T^, were evaluated under identical conditions at 30 °C. Carbon-source utilization was evaluated in liquid NMS medium supplemented with methanol, ethanol, or formaldehyde at 0.1% (v/v) or with formate, fructose, galactose, glucose, or xylose at 0.1% (w/v). Methanol tolerance was assessed in liquid NMS medium containing 0.2, 0.5, 1, 2, 3, 4, or 5% (v/v) methanol. Nitrogen-source utilization was evaluated by replacing KNO_3_ in liquid NMS medium with NH_4_Cl, urea, peptone, yeast extract, glycine, serine, valine, asparagine, aspartic acid, or glutamic acid at a final concentration of 0.05% (w/v). Gram staining was performed using a commercial kit (bioMérieux, France) according to the manufacturer’s instructions. Catalase activity was assessed by observing bubble production after exposure to 3% (v/v) hydrogen peroxide (Junsei, Japan), and oxidase activity was determined using 1% (w/v) tetramethyl-*p*-phenylenediamine (Merck, USA), as described by Smibert and Krieg (1994).

### 2.8 Cellular fatty acid analysis

Strains BW4-1^T^, SPW-1^T^, and PHL2-19^T^, and the reference strain were cultured in NMS medium at 30 °C and harvested during the exponential growth phase. Cellular fatty acids were extracted, saponified, and methylated according to the standard MIDI protocol (Sasser, 1990). The resulting fatty acid methyl esters were analyzed using a Hewlett-Packard 6,890 gas chromatograph and identified using the RTSBA6 database of the Sherlock Microbial Identification System (version 6.0B).

## 3 Results and discussion

### 3.1 Isolation of methanotrophic bacteria and phylogenetic characterization based on 16S rRNA gene sequences

Following enrichment, three potentially novel methanotrophic strains, designated BW4-1^T^, SPW-1^T^, and PHL2-19^T^, were isolated from wetland sediments. Nearly complete 16S rRNA gene sequences were obtained for strains BW4-1^T^ (1,469 bp), SPW-1^T^ (1,475 bp), and PHL2-19^T^ (1,476 bp) by assembling reads generated using primers 340F, 518R, and 805F from PCR amplicons produced with primers 27F and 1492R. Pairwise sequence comparisons showed that the three strains shared 99.9–100.0% 16S rRNA gene sequence similarity with one another. Their closest relative was *Methylomonas defluvii* OY6^T^, with sequence similarities of 99.9, 99.9, and 100.0% to strains BW4-1^T^, SPW-1^T^, and PHL2-19^T^, respectively. These results indicate that the three strains are closely related to one another and to *M. defluvii* based on their 16S rRNA gene sequences.

ML analysis based on 16S rRNA gene sequences showed that strains BW4-1^T^, SPW-1^T^, and PHL2-19^T^ formed a cluster with *M. defluvii* within the genus *Methylomonas* (Figure 1). NJ and MP analyses also supported this phylogenetic placement (Supplementary Figure S1). Collectively, the sequence comparisons and phylogenetic analyses indicate that strains BW4-1^T^, SPW-1^T^, and PHL2-19^T^ belong to the genus *Methylomonas* within the family *Methylococcaceae*.

**Figure 1:**
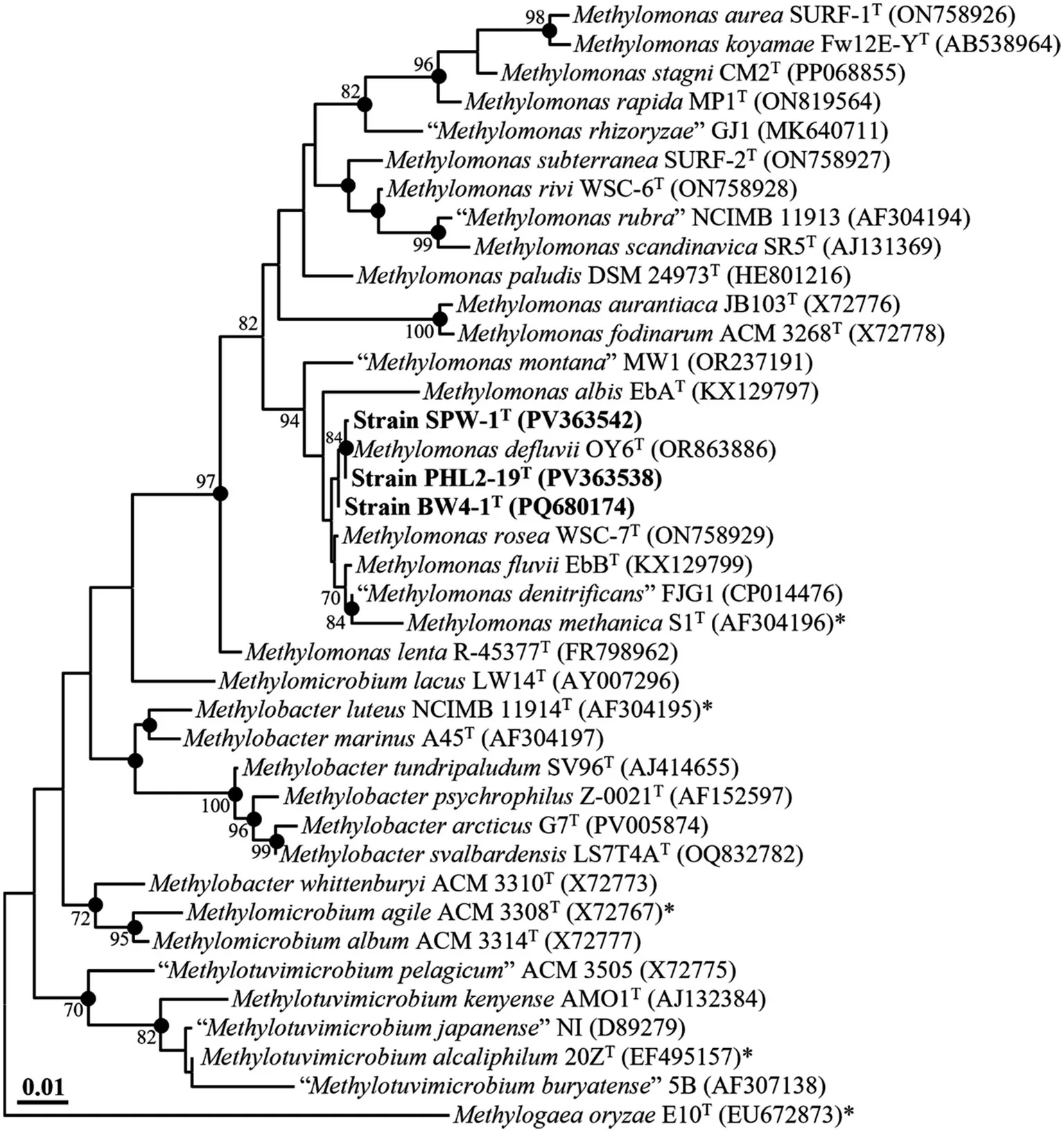
Maximum-likelihood tree based on 16S rRNA gene sequences, showing the phylogenetic relationships of strains BW4-1^T^, SPW-1^T^, and PHL2-19^T^ with closely related taxa. The type species of each genus are indicated by asterisks (*). Bootstrap values ≥70% based on 1,000 replicates are shown at the nodes. Filled circles (•) indicate nodes also recovered in the neighbor-joining and maximum-parsimony trees. *Methylogaea oryzae* E10^T^ (EU672873) was used as the outgroup. The scale bar represents 0.01 substitutions per nucleotide position.

### 3.2 Potential ecological distribution of strains BW4-1^T^, SPW-1^T^, and PHL2-19^T^ inferred using the IMNGS platform

The IMNGS platform is widely used to estimate the potential ecological distribution of microorganisms by comparing query 16S rRNA gene sequences against publicly available amplicon datasets (Lagkouvardos et al., 2016; Bayburt et al., 2026). However, the taxonomic resolution provided by 16S rRNA gene sequences varies considerably among phylogenetic groups, potentially biasing distribution estimates. This limitation is particularly relevant to *Methylomonas*, because many species within the genus share highly similar 16S rRNA gene sequences. Therefore, analyses based on a 99.0% similarity threshold may overestimate the distributions of individual species or strains.

Strains BW4-1^T^, SPW-1^T^, and PHL2-19^T^ shared 99.9–100.0% 16S rRNA gene sequence similarity with one another and consequently exhibited broadly similar distribution patterns in the IMNGS analysis (Supplementary Table S1). Moreover, because most recognized *Methylomonas* species share >99.0% 16S rRNA gene sequence similarity, the detected sequences cannot be reliably assigned to the three strains. Thus, the results should be interpreted as representing the potential ecological distribution of the genus *Methylomonas* rather than the strain- or species-specific distributions of BW4-1^T^, SPW-1^T^, and PHL2-19^T^.

At the 99.0% similarity threshold, *Methylomonas*-related sequences were detected across diverse aquatic, terrestrial, and host-associated datasets (Supplementary Table S1). Among the categories represented by at least 10 datasets, relatively high prevalence was observed in datasets associated with several beetle species, although these estimates were based on only one or two positive datasets per host species. The sequences were also detected frequently in frog-associated, aquifer, groundwater, root, freshwater, aquatic, wetland, sediment, and riverine datasets. Overall, the frequent detection of *Methylomonas*-related sequences in water-associated and water-saturated environments is consistent with previous reports that members of this genus commonly inhabit freshwater systems, sediments, wetlands, groundwater, and saturated soils (Deng et al., 2024). Nevertheless, their average relative abundances were consistently low (<0.06%), suggesting that *Methylomonas* generally constitutes a minor component of the microbial communities in these environments.

### 3.3 Genome sequencing, genome-based phylogeny, and genomic relatedness

*De novo* assembly of the MinION reads yielded complete genomes of 5,477 and 4,976 kb for strains BW4-1^T^ and SPW-1^T^, respectively, and a draft genome of 5,256 kb for strain PHL2-19^T^, with corresponding sequencing depths of 178×, 80×, and 131×, respectively. The 16S rRNA gene sequences extracted from the assembled genomes were identical to those obtained by PCR-based sequencing, confirming the consistency of the sequencing results. CheckM2 estimated 100% genome completeness for all three strains, with contamination levels of 4.3, 1.2, and 0.4% for strains BW4-1^T^, SPW-1^T^, and PHL2-19^T^, respectively. Thus, all three genomes met the generally accepted criteria for high-quality genomes (≥90% completeness and ≤10% contamination) (Chklovski et al., 2023). ML phylogenomic analysis based on the concatenated sequences of 120 conserved bacterial marker proteins showed that strains BW4-1^T^, SPW-1^T^, and PHL2-19^T^ clustered with *M. defluvii* OY6^T^ within the genus *Methylomonas* (Figure 2). This placement was consistent with the 16S rRNA gene-based phylogeny and confirmed the assignment of the three strains to the genus *Methylomonas*.

**Figure 2:**
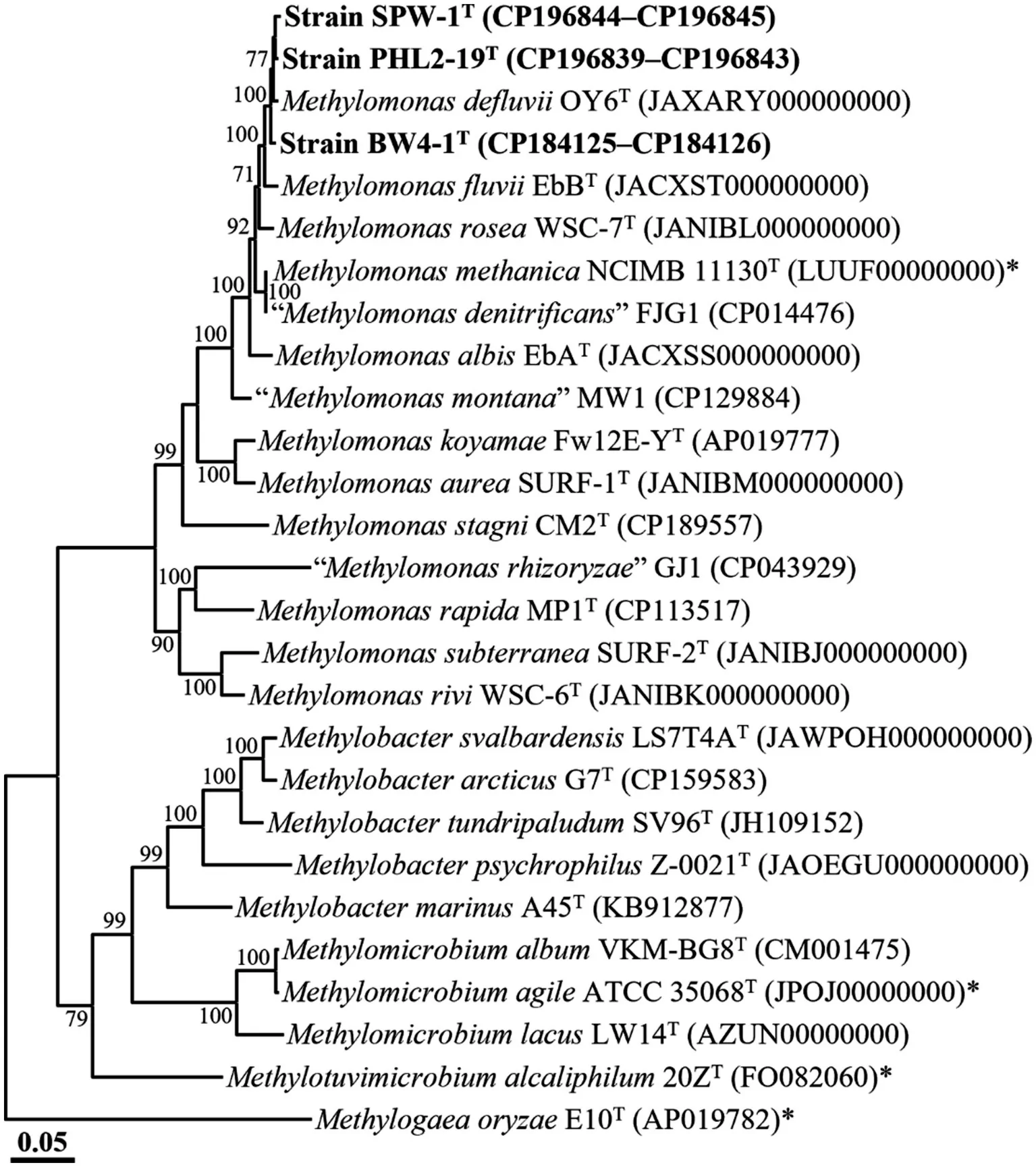
Maximum-likelihood phylogenomic tree based on the concatenated amino acid sequences of 120 conserved bacterial marker proteins (the bac120 marker set) identified using GTDB-Tk, showing the relationships of strains BW4-1^T^, SPW-1^T^, and PHL2-19^T^ with closely related taxa. Bootstrap values ≥70% based on 1,000 replicates are shown at the nodes. The type species of each genus are indicated by asterisks (*). *Methylogaea oryzae* E10^T^ (AP019782) was used as the outgroup. The scale bar represents 0.05 substitutions per amino acid position.

The ANI and dDDH values were 95.1 and 60.8% between BW4-1^T^ and SPW-1^T^, 95.0 and 60.4% between BW4-1^T^ and PHL2-19^T^, and 95.9 and 66.2% between SPW-1^T^ and PHL2-19^T^, respectively (Supplementary Table S2). Although the ANI values were within the proposed species-boundary range of 94–96%, all dDDH values were below the 70% threshold for species delineation (Riesco and Trujillo, 2024), supporting the recognition of the three strains as distinct species. The ANI values between strains BW4-1^T^, SPW-1^T^, and PHL2-19^T^ and their closest relative, *M. defluvii* OY6^T^, were 95.2, 95.8, and 95.7%, respectively, whereas the corresponding dDDH values were 61.1, 65.5, and 63.8%, further distinguishing the three strains from *M. defluvii*. Their ANI and dDDH values with all other type strains were well below the respective thresholds for species delineation. Collectively, the phylogenomic and genomic relatedness analyses indicate that strains BW4-1^T^, SPW-1^T^, and PHL2-19^T^ represent three distinct novel species of the genus *Methylomonas*.

### 3.4 Genomic features and phylogeny based on PmoA and MmoX

The genome sequences of strains BW4-1^T^, SPW-1^T^, and PHL2-19^T^ were deposited in GenBank and annotated using the NCBI Prokaryotic Genome Annotation Pipeline. The genome of strain BW4-1^T^ comprised a circular chromosome of 5,424 kb with a G + C content of 51.8 mol% and a circular plasmid of 53 kb with a G + C content of 50.8 mol%. The genome contained 5,109 genes, including 4,978 protein-coding sequences (CDSs), 47 tRNA genes collectively representing all 20 amino acids, three rRNA operons, each comprising 16S, 23S, and 5S rRNA genes, and four non-coding RNA genes.

The genome of strain SPW-1^T^ comprised a circular chromosome of 4,909 kb with a G + C content of 51.9 mol% and a circular plasmid of 67 kb with a G + C content of 47.8 mol%. It contained 4,599 genes, including 4,505 CDSs, 46 tRNA genes, three rRNA operons, and four non-coding RNA genes. The draft genome of strain PHL2-19^T^ consisted of five contigs with a total length of 5,256 kb and contained 4,877 genes, including 4,771 CDSs, 47 tRNA genes, three rRNA operons, and four non-coding RNA genes. The overall genomic G + C contents of strains BW4-1^T^, SPW-1^T^, and PHL2-19^T^ were 51.8, 51.9, and 51.9 mol%, respectively, consistent with the values reported for other *Methylomonas* species (Ouyang et al., 2024). Overall, the genome sizes, gene contents, and G + C contents of the three strains were comparable to those of closely related *Methylomonas* species (Table 1).

**Table 1:** General genomic features of strains BW4-1^T^, SPW-1^T^, and PHL2-19^T^ and their closest phylogenetic relative, *Methylomonas defluvii* OY6^T^.

Feature	1	2	3	4
Genome status (no. of contigs)^a^	C (2)	C (2)	D (5)	D (62)
Total genome size (kb)	5,477	4,976	5,256	5,414
G + C content (mol%)	51.8	51.9	51.9	51.7
No. of total genes	5,109	4,599	4,877	4,920
No. of protein coding sequences	4,978	4,505	4,771	4,818
No. of total RNA genes	60	59	60	49
No. of tRNA genes	47	46	47	42
No. of rRNA operons (16S, 23S, 5S)	3	3	3	1
No. of non-coding RNA genes	4	4	4	4
No. of pseudogenes	71	35	46	53

Taxa: 1, strain BW4-1^T^ (CP184125–CP184126); 2, strain SPW-1^T^ (CP196844–CP196845); 3, strain PHL2-19^T^ (CP196839–CP196843); 4, Methylomonas defluvii OY6^T^ (JAXARY000000000).

a C, complete; D, draft.

Members of the genus *Methylomonas* generally possess the *pmoCAB* gene cluster encoding pMMO, which catalyzes the initial oxidation of methane to methanol, whereas the *mmo* gene cluster encoding sMMO occurs only in some species or strains (Oshkin et al., 2023; Ouyang et al., 2024). To investigate the evolutionary relationships of these methane monooxygenases, ML phylogenetic analyses were performed using the amino acid sequences of PmoA and MmoX (Figure 3). The topology of the PmoA tree was broadly congruent with that of the genome-based phylogenomic tree (Figures 2, 3A). Strains BW4-1^T^, SPW-1^T^, and PHL2-19^T^ clustered within the *Methylomonas* lineage, and members of *Methylomonas*, *Methylobacter*, *Methylomicrobium*, and *Methylotuvimicrobium* formed genus-specific lineages. This broad congruence between the PmoA and genome-based phylogenies is consistent with the predominantly vertical inheritance of the *pmoCAB* gene cluster during the evolution of these methanotrophs.

**Figure 3:**
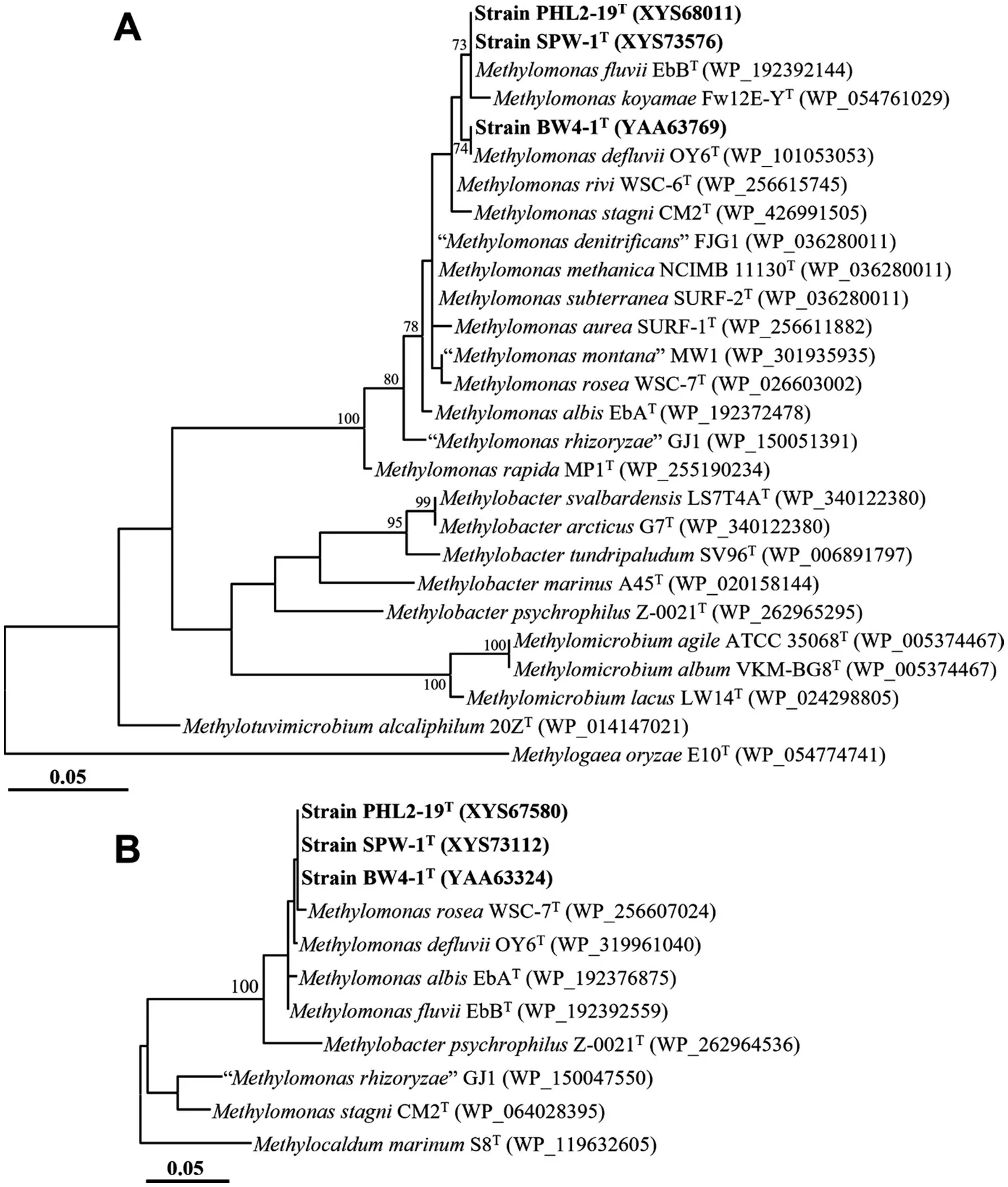
Maximum-likelihood trees based on the amino acid sequences of PmoA **(A)** and MmoX **(B)**, showing the phylogenetic relationships of strains BW4-1^T^, SPW-1^T^, and PHL2-19^T^ with closely related taxa. Bootstrap values >70% based on 1,000 replicates are shown at the nodes. *Methylogaea oryzae* E10^T^ (WP_054774741) and *Methylocaldum marinum* S8T (WP_119632605) were used as the outgroups in panels **(A,B)**, respectively. The scale bars represent 0.05 substitutions per amino acid position.

In contrast, MmoX was detected in only a subset of the analyzed taxa. In addition to strains BW4-1^T^, SPW-1^T^, and PHL2-19^T^, MmoX was identified in six *Methylomonas* species and one *Methylobacter* species (Figure 3B). The MmoX sequences of *Methylomonas* did not form a single monophyletic lineage: most clustered with *Methylobacter psychrophilus*, whereas those of “*Methylomonas rhizoryzae*” GJ1 and *Methylomonas stagni* CM2^T^ formed a separate lineage. The patchy distribution of MmoX and the incongruence between the MmoX and genome-based phylogenies suggest that the evolutionary history of the sMMO gene cluster involved lineage-specific gene gain or loss and possibly horizontal gene transfer, in contrast to the predominantly vertical inheritance of *pmoCAB*.

### 3.5 Metabolic potential and evolutionary dynamics of methane oxidation and carotenoid biosynthesis

Comparative genomic reconstruction revealed a broadly conserved metabolic framework linking methane oxidation and carbon assimilation to carotenoid biosynthesis (Figure 4). The *pmoCAB* operon encoding pMMO was conserved across all examined genomes, whereas the *mmoXYBZDC* cluster encoding sMMO showed a restricted distribution. Genes involved in methane oxidation by pMMO, methanol oxidation, formaldehyde assimilation through the ribulose monophosphate pathway, central carbon metabolism through the Entner-Doudoroff (ED) and Embden-Meyerhof-Parnas (EMP) pathways, and isoprenoid precursor formation through the methylerythritol phosphate (MEP) pathway were conserved across all analyzed genomes (Figure 4). Together, these pathways provide a metabolic route for converting methane-derived carbon into farnesyl diphosphate and geranylgeranyl diphosphate, the respective precursors of C_30_ and C_40_ carotenoids. A complete C_30_ diapocarotenoid pathway comprising *crtM*, *crtN*, *crtP*, and *crtNc* was present in all examined genomes, including those of the three novel strains, except *Methylobacter psychrophilus* Z-0021^T^ (Figure 5). This pathway mediates the sequential conversion of farnesyl diphosphate to 4,4′-diapophytoene, 4,4′-diapolycopene, 4,4′-diapolycopenedial, and 4,4′-diapolycopene-4,4′-dioic acid. This predicted capacity is consistent with the previous identification of 4,4′-diapolycopene-4,4′-dioic acid as a major carotenoid in *Methylomonas* and with the reddish-pink pigmentation of the three novel strains (Tikhonova et al., 2023; Table 2). A complete C_40_ carotenoid branch comprising *crtE*, *crtB*, and *crtI* was also detected in all genomes except those of three *Methylobacter* species: *Mb. svalbardensis* LS7T4A^T^, *Mb. psychrophilus* Z-0021^T^, and *Mb. marinus* A45^T^. This pathway directs geranylgeranyl diphosphate toward phytoene and lycopene biosynthesis. Thus, the genomic potential for both C_30_ and C_40_ carotenoid biosynthesis was highly conserved in *Methylomonas* but more variable within *Methylobacter*.

**Figure 4:**
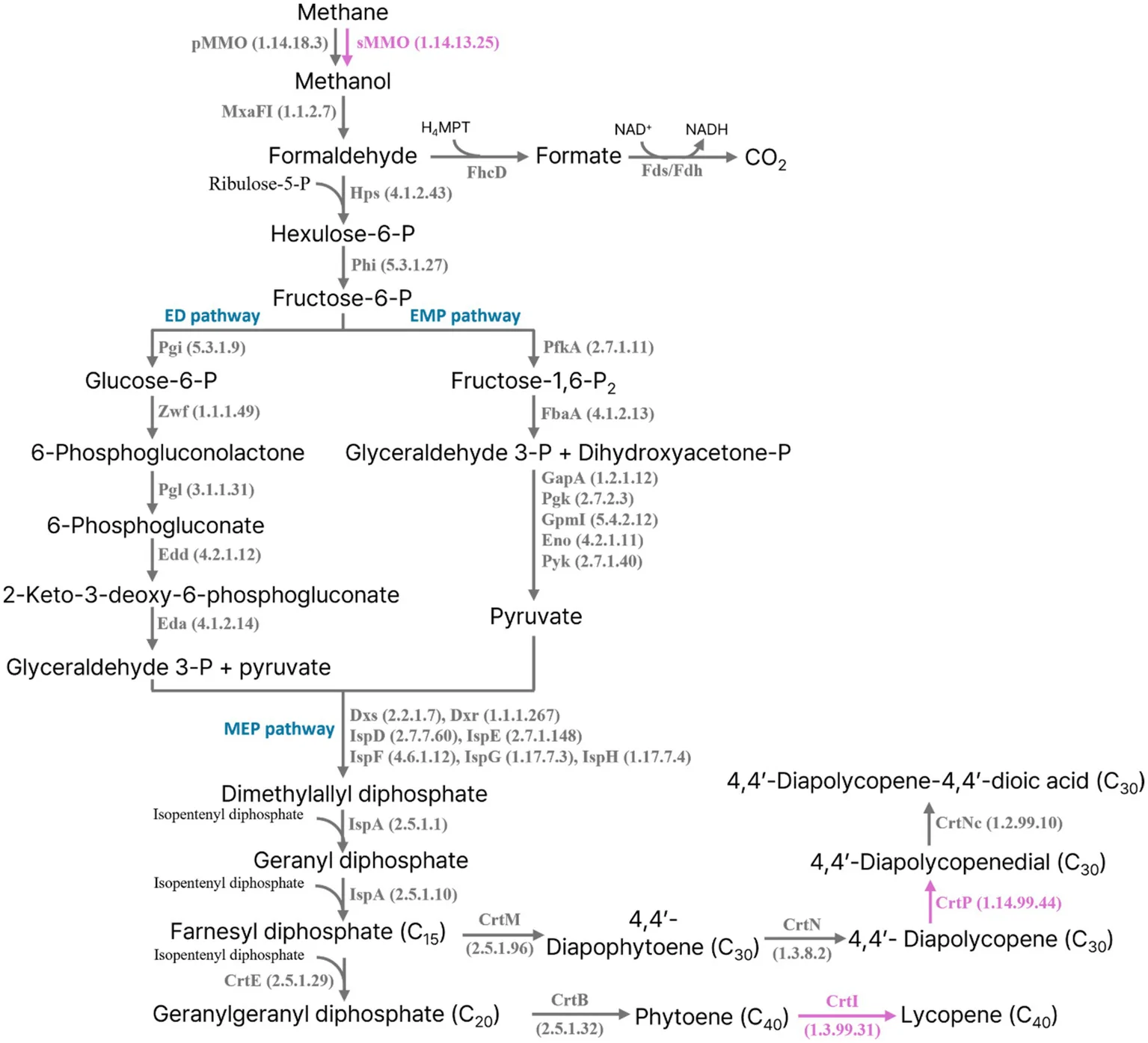
Predicted metabolic pathways linking methane oxidation and carbon assimilation to carotenoid biosynthesis in *Methylomonas* species and closely related taxa. Genes conserved across all analyzed genomes are shown in gray, whereas genes with variable distributions are shown in pink. Numbers in parentheses following gene names indicate Enzyme Commission (EC) numbers. Abbreviations: pMMO, particulate methane monooxygenase; sMMO, soluble methane monooxygenase; MxaFI, methanol dehydrogenase; FhcD, formyltransferase/hydrolase complex subunit D; Fds/Fdh, formate dehydrogenase; Hps, 3-hexulose-6-phosphate synthase; Phi, 6-phospho-3-hexuloisomerase; Pgi, glucose-6-phosphate isomerase; Zwf, glucose-6-phosphate dehydrogenase; Pgl, 6-phosphogluconolactonase; Edd, phosphogluconate dehydratase; Eda, 2-dehydro-3-deoxyphosphogluconate aldolase; PfkA, phosphofructokinase; FbaA, fructose-bisphosphate aldolase; GapA, glyceraldehyde-3-phosphate dehydrogenase; Pgk, phosphoglycerate kinase; GpmI, phosphoglycerate mutase; Eno, enolase; Pyk, pyruvate kinase; Dxs, 1-deoxy-d-xylulose-5-phosphate synthase; Dxr, 1-deoxy-d-xylulose-5-phosphate reductoisomerase; IspD, 2-C-methyl-d-erythritol 4-phosphate cytidylyltransferase; IspE, 4-diphosphocytidyl-2-C-methyl-d-erythritol kinase; IspF, 2-C-methyl-d-erythritol 2,4-cyclodiphosphate synthase; IspG, 4-hydroxy-3-methylbut-2-en-1-yl diphosphate synthase; IspH, 4-hydroxy-3-methylbut-2-en-1-yl diphosphate reductase; IspA, farnesyl diphosphate synthase; CrtE, geranylgeranyl diphosphate synthase; CrtM, 4,4′-diapophytoene synthase; CrtN, 4,4′-diapophytoene desaturase; CrtP, diapolycopene oxygenase; CrtNc, 4,4′-diapolycopenoate synthase; CrtB, phytoene synthase; and CrtI, phytoene desaturase.

**Figure 5:**
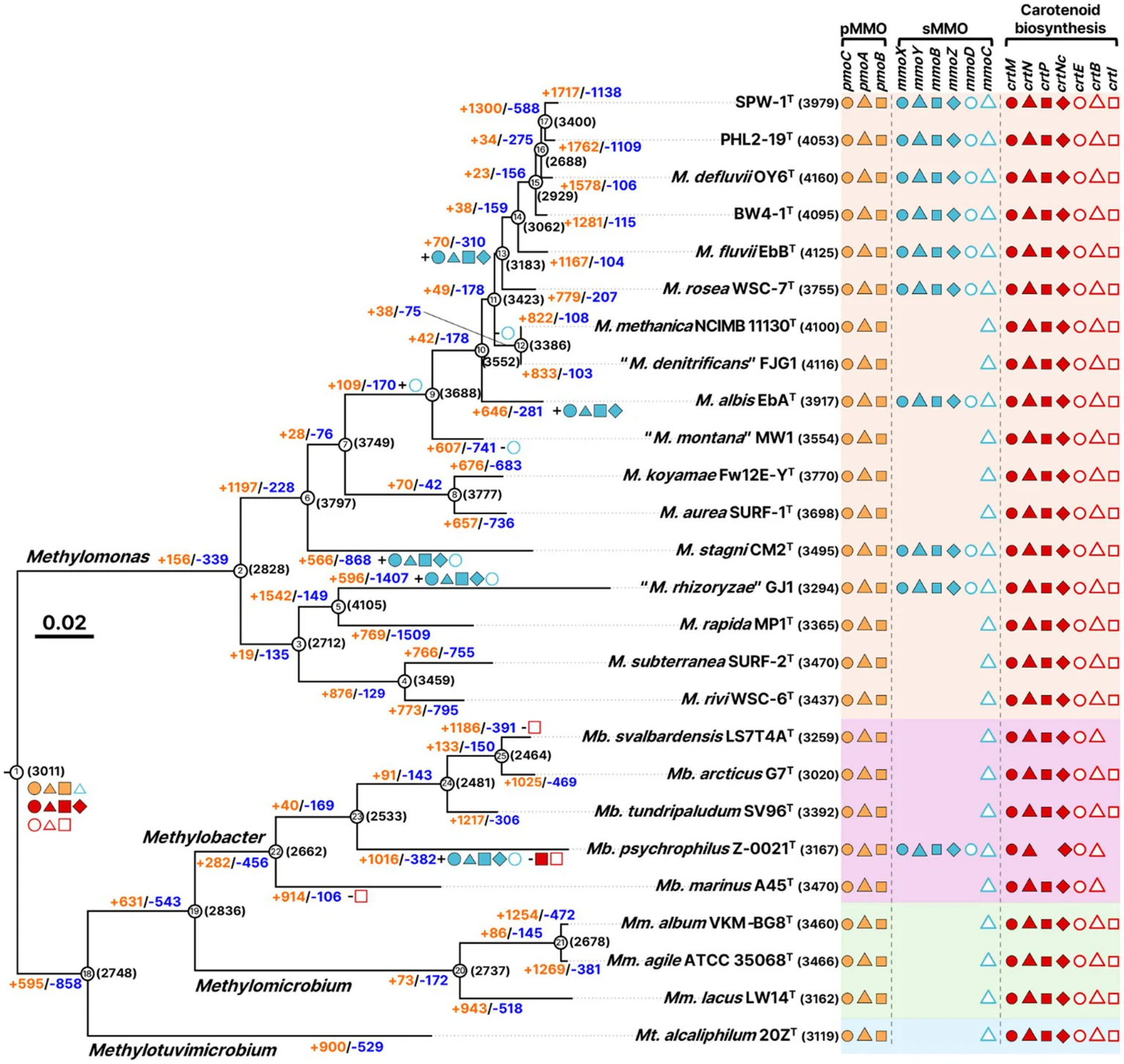
Ancestral reconstruction of gene gains and losses across the phylogeny of *Methylomonas* species and closely related taxa. Numbers along the branches indicate the inferred numbers of orthogroups gained (+; orange) and lost (−; blue). Numbers at internal nodes indicate the estimated sizes of ancestral orthogroup repertoires, whereas numbers in parentheses after taxon names indicate the numbers of orthogroups identified in extant genomes. Inferred gains (+) and losses (−) of representative genes involved in methane oxidation and carotenoid biosynthesis are indicated by the corresponding symbols at the nodes. The matrix to the right of the tree shows the distribution of these genes among extant genomes, with each symbol denoting the presence of the corresponding gene. Genera are distinguished by different background colors. Gene codes: *pmoA–C*, particulate methane monooxygenase; *mmoB*, *mmoC*, *mmoD*, *mmoX*, *mmoY*, and *mmoZ*, soluble methane monooxygenase; *crtE*, geranylgeranyl diphosphate synthase; *crtM*, 4,4′-diapophytoene synthase; *crtN*, 4,4′-diapophytoene desaturase; *crtP*, diapolycopene oxygenase; *crtNc*, 4,4′-diapolycopenoate synthase; *crtB*, phytoene synthase; and *crtI*, phytoene desaturase.

**Table 2:** Differential phenotypic characteristics between strains BW4-1^T^, SPW-1^T^, and PHL2-19^T^ and their closest phylogenetic relative, *Methylomonas defluvii*.

Characteristic	1	2	3	4
Geographic origin	Hwaseong, South Korea	Gangneung, South Korea	Gangneung, South Korea	Shanghai, China
Isolation source	Wetland sediment	Wetland sediment	Wetland sediment	Sedimentation tank of wastewater treatment plant
Growth range^a^ of:				
Temperature (°C, optimum)	15–30 (30)	10–35 (30)	15–35 (30)	20–37 (30)
pH (optimum)	6.5–8.0 (7.0)	6.0–8.0 (7.0)	6.5–7.5 (6.5)	4.5–7.5 (6.5)
NaCl (%, w/v, optimum)	0–0.5 (0)	0–0.5 (0.5)	0–0.5 (0)	0–0.5 (0)
Methanol (%, v/v)	0.2–4.0	0.2–4.0	0.2–3.0	NA
Activity^a^ of:				
Catalase	−	+	+	+
Oxidase	*w*	+	*w*	−
Other N sources^a^	Urea, NH_4_Cl, peptone, yeast extract, serine	Urea, NH_4_Cl, peptone, yeast extract, glycine, serine	Urea, NH_4_Cl, peptone, yeast extract, serine, valine	NH_4_Cl, yeast extract, peptone, casamino acids^b^, tryptophan^b^, serine, glutamine^b^

Taxa: 1, strain BW4-1^T^; 2, strain SPW-1^T^; 3, strain PHL2-19^T^; 4, *M. defluvii* KCTC 8159^T^ (Ouyang et al., 2024). All strains are rod-shaped type I methanotrophs that form reddish-pink colonies. Among the alternative carbon sources tested, only methanol supports growth; ethanol, formaldehyde, formate, fructose, galactose, glucose, and xylose are not utilized. All strains possess pmoA, mmoX, and nifH genes.

Symbols: +, positive; −, negative; w, weakly positive; NA, not available.

a These data were obtained under the same conditions in this study.

b The data were obtained from Ouyang et al. (2024).

Ancestral reconstruction revealed extensive genome-wide orthogroup turnover but contrasting evolutionary stability among genes involved in methane oxidation and carotenoid biosynthesis (Figure 5). The common ancestor of the analyzed taxa was inferred to contain 3,011 orthogroups, with the numbers of branch-specific gains and losses varying considerably among lineages. Particularly extensive turnover was inferred along the terminal branches leading to SPW-1^T^ and PHL2-19^T^, with 1,717 gains and 1,138 losses and 1,762 gains and 1,109 losses, respectively. The branch leading to BW4-1^T^ exhibited 1,281 gains and 115 losses. These results indicate substantial divergence in gene repertoires among *Methylomonas* species, including the three novel strains, despite their close phylogenetic relationships. Such extensive turnover likely reflects ongoing remodeling of the accessory genome and may underlie species-specific differences in metabolic capacity, stress responses, and adaptation to distinct ecological niches. Thus, phylogenetic similarity among species within the genus *Methylomonas* may not necessarily imply equivalent metabolic potential.

The extensive gene-repertoire turnover observed among these closely related *Methylomonas* species may help explain the apparent discrepancy between highly conserved phylogenomic markers and lower genome-wide relatedness. The 120 bacterial marker proteins, PmoA, and MmoX used for phylogenetic analyses are highly conserved among these species (Figures 2, 3), whereas ANI and dDDH reveal greater nucleotide-level divergence across broader homologous genomic regions. Thus, closely related species may retain highly similar conserved proteins while differing substantially in accessory-gene content and genome-wide nucleotide sequences. The extensive orthogroup gains inferred for strain BW4-1^T^, SPW-1^T^, and PHL2-19^T^ indicate substantial gene-repertoire remodeling, suggesting that accessory-genome divergence may contribute to their genomic differentiation. However, accessory-gene differences alone cannot explain the ANI values, which primarily reflect nucleotide identity within homologous genomic regions. Nucleotide substitutions within shared genomic regions, including synonymous substitutions that do not alter protein sequences, may therefore also contribute to the observed divergence. Nevertheless, the relative contributions of these processes cannot be resolved from the present analyses.

The common ancestor was inferred to possess the complete *pmoCAB* operon and all genes required for 4,4′-diapolycopene-4,4′-dioic acid and lycopene biosynthesis, most of which were retained across the phylogeny. This conservation indicates that pMMO-dependent methane oxidation and C_30_ and C_40_ carotenoid biosynthesis are ancestral and evolutionarily stable traits in these methanotrophs. The principal exceptions were lineage-specific losses of *crtP* and/or *crtI* in *Mb. svalbardensis* LS7T4A^T^, *Mb. psychrophilus* Z-0021^T^, and *Mb. marinus* A45^T^. These losses are predicted to truncate the corresponding carotenoid pathways and may contribute to differences in carotenoid composition and pigmentation within *Methylobacter*.

By contrast, *mmoC* was the only sMMO-associated gene inferred to have been present in the common ancestor, and an *mmoC* homolog was retained in every extant genome (Figure 5). Because *mmoC* encodes the reductase component that transfers electrons to the sMMO hydroxylase, its presence alone does not indicate sMMO activity, which requires the coordinated expression and assembly of the complete multicomponent system (Cheng et al., 2025). The universal retention of *mmoC* despite the frequent absence of other sMMO genes suggests that MmoC or MmoC-like reductases may have a conserved function independent of the complete sMMO system (Miao and Schmidt, 2025). Nevertheless, their physiological electron-transfer partners and potential roles in other redox processes remain unknown. Thus, an essential function cannot be inferred from gene distribution alone and requires further phylogenetic, genomic-context, expression, and biochemical analyses.

In contrast to the universal occurrence of *mmoC*, complete *mmoXYBZDC* clusters exhibited a patchy, species-specific distribution that was incongruent with genus-level phylogeny. Complete clusters were detected in nine of the 17 *Methylomonas* genomes, including BW4-1^T^, SPW-1^T^, and PHL2-19^T^, and in only one of the five *Methylobacter* genomes, but not in the examined *Methylomicrobium* or *Methylotuvimicrobium* genomes. Together with the inferred branch-specific gains and losses, this discontinuous distribution is more consistent with recurrent acquisition and loss, potentially involving horizontal gene transfer, than with strict vertical inheritance of an intact sMMO cluster. Acquisition and retention of complete sMMO systems may confer a selective advantage by enabling methane oxidation under copper-limited conditions, when the expression and activity of copper-dependent pMMO are restricted. The coexistence of complete pMMO and sMMO systems may therefore enhance the metabolic flexibility of BW4-1^T^, SPW-1^T^, and PHL2-19^T^ under fluctuating copper availability in wetland sediments (Kalyuzhnaya et al., 2025).

Nevertheless, the ancestral reconstruction has several limitations. Because the analysis was restricted to *Methylomonas* and the closely related genera *Methylobacter* and *Methylomicrobium*, the inferred evolutionary patterns should be interpreted within this phylogenetic framework and may not represent trait evolution across the entire family *Methylococcaceae*. In addition, the ancestral gene contents and gain/loss events inferred using Wagner parsimony depend on taxon sampling, orthogroup assignments, the reference phylogeny, and the assumptions of the reconstruction method. Therefore, these results should be regarded as evolutionary inferences rather than definitive reconstructions of individual ancestral states or gain/loss events.

### 3.6 Physiological, phenotypic, and biochemical characteristics

Cells of strains BW4-1^T^, SPW-1^T^, and PHL2-19^T^ were Gram-stain-negative, motile, flagellated short rods measuring 0.6–1.1 × 1.0–1.7 μm, 0.7–1.1 × 0.9–1.5 μm, and 0.8–1.1 × 1.1–1.4 μm, respectively (Figure 6). All three strains contained bright intracellular granules resembling the polyhydroxyalkanoate storage granules reported in methanotrophic bacteria (Graham et al., 1991). They also possessed intracytoplasmic membranes arranged as stacks of vesicular discs perpendicular to the cytoplasmic membrane, a characteristic feature of type I methanotrophs that supports pMMO-dependent methane oxidation (Figure 6; Ouyang et al., 2024). All three strains grew with methane or methanol as the sole carbon and energy source. Strains BW4-1^T^ and SPW-1^T^ grew at methanol concentrations of 0.2–4.0% (v/v), whereas PHL2-19^T^ grew at concentrations of 0.2–3.0% (v/v).

**Figure 6:**
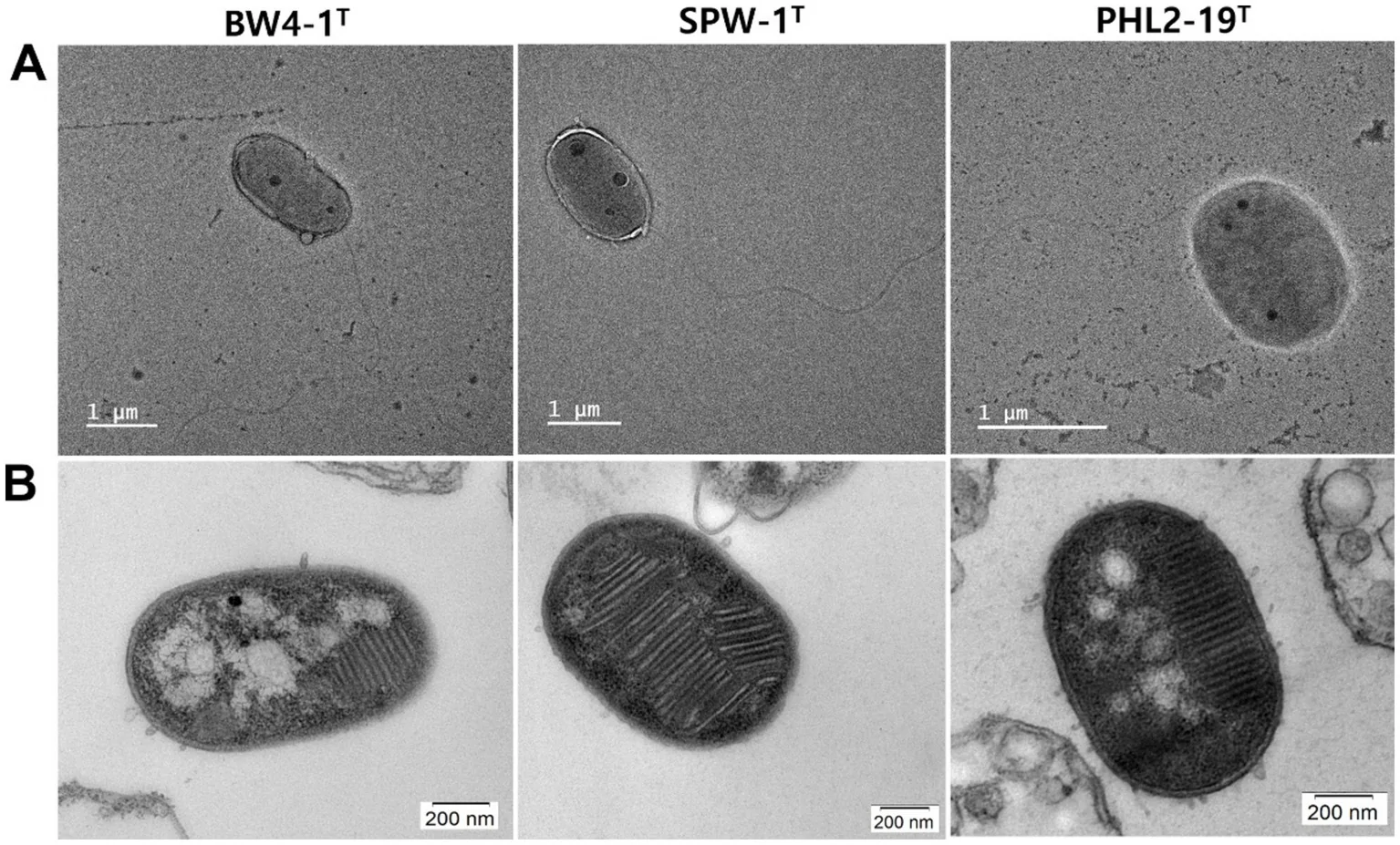
Transmission electron micrographs of strains BW4-1^T^, SPW-1^T^, and PHL2-19^T^ cultured on NMS agar at 30 °C for 2 days. **(A)** Cells negatively stained with UranyLess. **(B)** Ultrathin sections double-stained with 2% uranyl acetate and 3% lead citrate.

All three strains utilized urea, NH_4_Cl, peptone, yeast extract, and serine as nitrogen sources. In addition, strain SPW-1^T^ utilized glycine, whereas strain PHL2-19^T^ utilized valine (Table 2). The genomes of all three strains contained *nifH*, which encodes the nitrogenase Fe-protein subunit involved in biological nitrogen fixation. The occurrence of *nifH* and nitrogenase activity has also been reported in diverse type I and type II methanotrophs, including members of *Methylobacter* and *Methylomonas* (Auman et al., 2001). More recently, nitrogen fixation associated with methane cycling has been demonstrated in groundwater microbial communities dominated by methanotrophic *Methylomonadaceae* (Li et al., 2025). These findings suggest that strains BW4-1^T^, SPW-1^T^, and PHL2-19^T^ may possess the genomic potential for nitrogen fixation, which could provide an ecological advantage in nitrogen-limited wetland sediments. However, because the presence of *nifH* alone does not demonstrate a functional nitrogenase system, this potential should be confirmed experimentally.

### 3.7 Cellular fatty acid composition

The major fatty acids (>10.0%) of strains BW4-1^T^, SPW-1^T^, and PHL2-19^T^ were C_14:0_, C_16:0_, and summed feature 3 (C_16:1_ *ω*7*c* and/or C_16:1_ *ω*6*c*) (Supplementary Table S3), consistent with profiles reported for closely related *Methylomonas* species (Ogiso et al., 2012; Hoefman et al., 2014; Bussmann et al., 2021; Tikhonova et al., 2023; Abin et al., 2024; Gwak et al., 2025). The three strains and *M. defluvii* KCTC 8159^T^ shared broadly similar profiles but differed substantially in the relative abundances of several components. Summed feature 3 was particularly abundant in BW4-1^T^ and PHL2-19^T^ (62.9 and 56.1%, respectively), whereas SPW-1^T^ contained a higher proportion of C_16:0_ (36.2%). C_18:0_ was absent from BW4-1^T^ and occurred at lower levels in SPW-1^T^ and PHL2-19^T^ than in *M. defluvii* KCTC 8159^T^, while C_18:1_ *ω*9*c* was detected only in the reference strain. Strain-dependent differences were also observed in the minor fatty acids iso-C_17:0_, anteiso-C_16:0_, and C_16:0_ 3-OH. These differences further distinguish the three novel strains from one another and from *M. defluvii*.

## 4 Conclusion

Three reddish-pink, type I methanotrophs, designated BW4-1^T^, SPW-1^T^, and PHL2-19^T^, were isolated from wetland sediments in the Republic of Korea. Although their 16S rRNA gene sequences were nearly identical to one another and to that of *Methylomonas defluvii*, phylogenomic placement, ANI and dDDH values, and phenotypic and chemotaxonomic differences demonstrated that they represent three distinct species of the genus *Methylomonas*. Comparative genomic analyses showed that all three strains possess complete *pmoCAB* and *mmoXYBZDC* clusters, indicating the genomic capacity for both particulate and soluble methane monooxygenase systems. They also harbor conserved pathways linking methane-derived carbon to carotenoid biosynthesis, consistent with their reddish-pink pigmentation. Across *Methylomonas* and related genera, pMMO-dependent methane oxidation and carotenoid biosynthetic capacity were inferred to be ancestral and largely conserved, whereas sMMO-associated genes exhibited lineage-specific gain, loss, and possible horizontal transfer. These findings expand the taxonomic diversity of *Methylomonas* and provide insights into the evolution of methane oxidation and carotenoid biosynthesis in type I methanotrophs.

## 5 Taxonomic descriptions

Phylogenetic, phylogenomic, and genomic relatedness analyses demonstrated that the three isolates represent distinct novel species of the genus *Methylomonas* (Figures 1, 2, Supplementary Figure S1, and Supplementary Table S2). They were further differentiated by their phenotypic and biochemical characteristics (Table 2) and cellular fatty acid profiles (Supplementary Table S3). Accordingly, the names *Methylomonas sediminis* sp. nov., *Methylomonas paludicola* sp. nov., and *Methylomonas roseola* sp. nov. are proposed for strains BW4-1^T^, SPW-1^T^, and PHL2-19^T^, respectively.

### 5.1 Description of *Methylomonas sediminis* sp. nov

*Methylomonas sediminis* (se.di′mi.nis. N.L. gen. n. *sediminis*, of sediment).

Colonies grown on NMS medium are reddish pink, circular, and smooth. Cells are Gram-stain-negative, strictly aerobic, motile, flagellated rods. Oxidase activity is weakly positive, whereas catalase activity is negative. Growth occurs at 15–30 °C (optimum, 30 °C), at pH 6.5–8.0 (optimum, pH 7.0), and with 0–0.5% (w/v) NaCl (optimum, 0%). Only methane and methanol support growth; ethanol, formaldehyde, formate, fructose, galactose, glucose, and xylose are not utilized. The strain uses urea, NH_4_Cl, peptone, yeast extract, and serine as nitrogen sources. The genes *pmoA*, *mmoX*, and *nifH* are detected in the genome. The major cellular fatty acids (>10.0%) are C_14:0_, C_16:0_, and summed feature 3 (C_16:1_ *ω*7*c* and/or C_16:1_ *ω*6*c*).

The type strain, BW4-1^T^ (=KACC 24053^T^ = MCCC 1K10705^T^), was isolated from wetland sediment in Hwaseong-si, Republic of Korea. Its genome is 5,477 kb in size and has a DNA G + C content of 51.8 mol%. The GenBank accession numbers for the 16S rRNA gene and complete genome sequences are PQ680174 and CP184125–CP184126, respectively.

### 5.2 Description of *Methylomonas paludicola* sp. nov

*Methylomonas paludicola* (pa.lu.di′co.la. L. neut. n. *palus*, a marsh, swamp; L. suffix -*cola* (from L. masc. or fem. n. *incola*), inhabitant, dweller; N.L. fem. n. *paludicola*, a marsh dweller).

Colonies grown on NMS medium are reddish pink, circular, and smooth. Cells are Gram-stain-negative, strictly aerobic, motile, flagellated rods. Oxidase and catalase activities are positive. Growth occurs at 10–35 °C (optimum, 30 °C), at pH 6.0–8.0 (optimum, pH 7.0), and with 0–0.5% (w/v) NaCl (optimum, 0.5%). Only methane and methanol support growth; ethanol, formaldehyde, formate, fructose, galactose, glucose, and xylose are not utilized. The strain uses urea, NH_4_Cl, peptone, yeast extract, glycine, and serine as nitrogen sources. The genes *pmoA*, *mmoX*, and *nifH* are detected in the genome. The major cellular fatty acids (>10.0%) are C_14:0_, C_16:0_, and summed feature 3 (C_16:1_ *ω*7*c* and/or C_16:1_ *ω*6*c*).

The type strain, SPW-1^T^ (=KACC 24052^T^ = MCCC 1K10729^T^), was isolated from wetland sediment in Gangneung-si, Republic of Korea. Its genome is 4,976 kb in size and has a DNA G + C content of 51.9 mol%. The GenBank accession numbers for the 16S rRNA gene and complete genome sequences are PV363542 and CP196844–CP196845, respectively.

### 5.3 Description of *Methylomonas roseola* sp. nov

*Methylomonas roseola* (ro.se′o.la. N.L. fem. Adj. *roseola*, light pink, rose pink).

Colonies on NMS medium are reddish pink, circular, and smooth. Cells are Gram-stain-negative, strictly aerobic, flagellated motile rods. Oxidase-weakly positive and catalase-positive. Growth occurs at 15–35 °C (optimum, 30 °C), at pH 6.5–7.5 (optimum, pH 6.5), and in the presence of 0–0.5% (w/v) NaCl (optimum, 0%). Only methane and methanol support growth; ethanol, formaldehyde, formate, fructose, galactose, glucose, and xylose are not utilized. The strain uses urea, NH_4_Cl, peptone, yeast extract, serine, and valine as nitrogen sources. The genes *pmoA*, *mmoX*, and *nifH* are detected in the genome. Major cellular fatty acids (>10.0%) include C_14:0_, C_16:0_, and summed feature 3 (C_16:1_ *ω*7*c* and/or C_16:1_ *ω*6*c*).

The type strain, PHL2-19^T^ (=KACC 24054^T^ = MCCC 1K10706^T^), was isolated from wetland sediment in Gangneung-si, Republic of Korea. Its genome is 5,256 kb in size and has a DNA G + C content of 51.9 mol%. The GenBank accession numbers for the 16S rRNA gene and genome sequences are PV363538 and CP196839–CP196843, respectively.

## Data Availability

The datasets presented in this study can be found in online repositories. The names of the repository/repositories and accession number(s) can be found in the article. The sequence data presented in this study are deposited in the GenBank repository under accession numbers PQ680174, PV363542, PV363538, CP184125–CP184126, CP196844–CP196845, and CP196839– CP196843.

## Footnotes

1
https://www.uniprot.org
