# Supplementary material for: Methylomonassediminis sp. nov., Methylomonas paludicola sp. nov., and Methylomonas roseola sp. nov.: genomic insights into the evolution of methane oxidation and carotenoid biosynthesis
Source: Front Microbiol. 2026 Sep 22;17:1954800. doi: 10.3389/fmicb.2026.1954800 (PMC13638612; doi:10.3389/fmicb.2026.1954800)
Supplement: Supplementary file 1 [file Supplementary_file_1.DOCX]

**SUPPLEMENTARY MATERIAL**

**FIGURE S1.** Neighbor-joining (NJ; A) and maximum-parsimony (MP; B) trees based on 16S rRNA gene sequences, showing the phylogenetic relationships of strains BW4-1^T^, SPW-1^T^, and PHL2-19^T^ with closely related taxa. The type species of each genus are indicated by asterisks (*). Bootstrap values ≥70% based on 1,000 replicates are shown at the nodes. *Methylogaea oryzae* E10^T^ (EU672873) was used as the outgroup. The scale bar in the NJ tree represents nucleotide substitutions per site, whereas that in the MP tree represents the number of nucleotide changes across the entire 16S rRNA gene sequence.

**TABLE S1.** Potential ecological distributions of strains BW4-1^T^, SPW-1^T^ and PHL2-19^T^, assessed by comparing their 16S rRNA gene sequences against environmental 16S rRNA gene amplicon datasets using the Integrated Microbial Next-Generation Sequencing (IMNGS) platform at a sequence similarity threshold of 99.0%. “Matched no. (prevalence)” indicates the number and percentage, respectively, of datasets containing sequences that matched the 16S rRNA gene sequence of each strain, whereas “ARA” indicates the average relative abundance of the matching sequences within these datasets. Environmental categories represented by fewer than 10 datasets or with ARA values below 0.0001% for all three strains were excluded.

| **Metagenome datasets** | **No. of datasets** | **Strain BW4-1^T^** | | **Strain SPW-1^T^** | | **Strain PHL2-19^T^** | |
| --- | --- | --- | --- | --- | --- | --- | --- |
|  |  | **Matched**  **no. (prevalence)** | **ARA (%)** | **Matched**  **no. (prevalence)** | **ARA (%)** | **Matched**  **no. (prevalence)** | **ARA**  **(%)** |
| *Aphthona venustula* | 10 | 2 (20.0) | 8.64E-03 | 2 (20.0) | 8.64E-03 | 2 (20.0) | 8.64E-03 |
| *Eusomus ovulum* | 10 | 2 (20.0) | 3.11E-03 | 2 (20.0) | 3.11E-03 | 2 (20.0) | 3.11E-03 |
| *Paederidus ruficollis* | 10 | 2 (20.0) | 2.01E-03 | 2 (20.0) | 2.00E-03 | 2 (20.0) | 2.01E-03 |
| *Paederus limnophilus* | 10 | 2 (20.0) | 2.03E-04 | 2 (20.0) | 2.00E-04 | 2 (20.0) | 2.03E-04 |
| *Proteobacteria bacterium* | 18 | 2 (11.1) | 4.22E-05 | 2 (11.1) | 1.23E-02 | 2 (11.1) | 4.22E-05 |
| *Bembidion punctulatum* | 10 | 1 (10.0) | 1.06E-03 | 1 (10.0) | 1.06E-03 | 1 (10.0) | 1.06E-03 |
| *Bembidion varicolor* | 10 | 1 (10.0) | 1.82E-03 | 1 (10.0) | 1.82E-03 | 1 (10.0) | 1.82E-03 |
| *Crioceris quatuordecimpunctata* | 10 | 1 (10.0) | 2.14E-03 | 1 (10.0) | 2.14E-03 | 1 (10.0) | 2.14E-03 |
| Frog metagenome | 168 | 14 (8.3) | 1.31E-02 | 14 (8.3) | 1.31E-02 | 14 (8.3) | 1.31E-02 |
| Aquifer metagenome | 237 | 16 (6.8) | 3.96E-03 | 16 (6.8) | 3.96E-03 | 16 (6.8) | 3.96E-03 |
| Groundwater metagenome | 745 | 40 (5.4) | 6.64E-03 | 40 (5.4) | 6.64E-03 | 40 (5.4) | 6.64E-03 |
| Plastisphere metagenome | 26 | 1 (3.9) | 3.99E-04 | 1 (3.9) | 4.00E-04 | 1 (3.9) | 3.99E-04 |
| Root metagenome | 2,024 | 76 (3.8) | 6.89E-03 | 76 (3.8) | 6.89E-03 | 76 (3.8) | 6.89E-03 |
| Freshwater metagenome | 14,593 | 500 (3.4) | 8.11E-03 | 512 (3.5) | 8.32E-03 | 512 (3.5) | 8.32E-03 |
| Biofilter metagenome | 181 | 5 (2.8) | 4.30E-04 | 5 (2.8) | 4.30E-04 | 5 (2.8) | 4.30E-04 |
| Industrial waste metagenome | 38 | 1 (2.6) | 1.97E-04 | 1 (2.6) | 2.00E-04 | 1 (2.6) | 1.97E-04 |
| Aquatic metagenome | 10,493 | 272 (2.6) | 1.47E-02 | 272 (2.6) | 1.48E-02 | 272 (2.6) | 1.48E-02 |
| Wetland metagenome | 80 | 6 (7.5) | 9.53E-04 | 2 (2.5) | 2.10E-04 | 6 (7.5) | 9.53E-04 |
| Sediment metagenome | 4,605 | 82 (1.8) | 8.11E-04 | 84 (1.8) | 8.20E-04 | 82 (1.8) | 8.19E-04 |
| Microbial mat metagenome | 720 | 1 (0.1) | 6.91E-07 | 13 (1.8) | 5.99E-02 | 13 (1.8) | 5.99E-02 |
| Riverine metagenome | 349 | 59 (16.9) | 3.19E-02 | 6 (1.7) | 1.80E-04 | 6 (1.7) | 1.84E-04 |
| Milk metagenome | 1,544 | 21 (1.4) | 1.15E-04 | 21 (1.4) | 1.10E-04 | 21 (1.4) | 1.15E-04 |
| Phyllosphere metagenome | 1,061 | 14 (1.3) | 5.63E-04 | 14 (1.3) | 5.60E-04 | 14 (1.3) | 5.63E-04 |
| Sludge metagenome | 1,924 | 20 (1.0) | 5.78E-04 | 20 (1.0) | 5.80E-04 | 20 (1.0) | 5.78E-04 |

**TABLE S2**. Pairwise digital DNA-DNA hybridization (dDDH) and average nucleotide identity (ANI) among strains BW4-1^T^, SPW-1^T^, and PHL2-19^T^ and their closest phylogenetic relative, *Methylomonas defluvii*.

Taxa: 1, strain BW4-1^T^ (CP184125–CP184126); 2, strain SPW-1^T^ (CP196844–CP196845); 3, strain PHL2-19^T^ (CP196839–CP196843); 4, *M. defluvii* OY6^T^ (JAXARY000000000)

|  | dDDH value (%) | | | | |
| --- | --- | --- | --- | --- | --- |
|  |  | **1** | **2** | **3** | **4** |
| ANI value (%) | **1** | – | 60.8 | 60.4 | 61.1 |
|  | **2** | 95.1 | – | 66.2 | 65.5 |
|  | **3** | 95.0 | 95.9 | – | 63.8 |
|  | **4** | 95.2 | 95.8 | 95.7 | – |

**TABLE S3.** Cellular fatty acid compositions (%) of BW4-1^T^, SPW-1^T^, and PHL2-19^T^ and their closest phylogenetic relative, *Methylomonas defluvii*.

Taxa: 1, strain BW4-1^T^; 2, strain SPW-1^T^; 3, strain PHL2-19^T^; 4, *M. defluvii* KCTC 8159^T^. All data were obtained in this study and are expressed as percentages of the total fatty acids. Fatty acids accounting for <1.0% in all strains are omitted. Major fatty acids (>10.0%) are shown in bold. Abbreviations: tr, trace amount (<1.0%); –, not detected.

| **Fatty acid** | 1 | 2 | 3 | 4 |
| --- | --- | --- | --- | --- |
| Saturated: |  |  |  |  |
| C_12:0_ | tr | 1.1 | tr | tr |
| C_14:0_ | **19.8** | **22.7** | **25.4** | **17.8** |
| C_16:0_ | **12.6** | **36.2** | **13.3** | **25.5** |
| C_18:0_ | – | 7.7 | tr | **18.9** |
| Unsaturated: |  |  |  |  |
| C_18:1_ *ω*9*c* | – | – | – | 2.4 |
| Branched: |  |  |  |  |
| iso-C_17:0_ | – | 1.4 | – | tr |
| anteiso-C_16:0_ | – | 1.7 | – | – |
| Hydroxy: |  |  |  |  |
| C_16:0_ 3-OH | 2.8 | – | 3.1 | 1.2 |
| Summed feature* |  |  |  |  |
| 3 (C_16:1_ *ω*7*c* and/or C_16:1_ *ω*6*c*) | **62.9** | **22.8** | **56.1** | **26.5** |
| 8 (C_18:1_ *ω*7*c* and/or C_18:1_ *ω*6*c*) | tr | – | tr | 3.0 |

*Summed features are a group of fatty acids that cannot be resolved reliably using the chromatographic conditions employed. The MIDI system classifies these fatty acids as one feature with a single percentage of the total.
